# Understanding Membership in Alternative Health Social Media Groups and Its Association with COVID-19 and Influenza Vaccination: Web-Based Cross-Sectional Survey

**DOI:** 10.2196/54092

**Published:** 2024-12-05

**Authors:** Kilhoe Na, Melissa Zimdars, Megan E Cullinan

**Affiliations:** 1 Department of Communication and Media Merrimack College North Andover, MA United States; 2 Department of Communication Studies University of Montana Missoula, MT United States

**Keywords:** alternative health, social media, misinformation, vaccination, COVID-19, Coronavirus

## Abstract

**Background:**

Social media platforms have become home to numerous alternative health groups where people share health information and scientifically unproven treatments. Individuals share not only health information but also health misinformation in alternative health groups on social media. Yet, little research has been carried out to understand members of these groups. This study aims to better understand various characteristics of members in alternative health groups and the association between membership and attitudes toward vaccination and COVID-19 and influenza vaccination–related behaviors.

**Objective:**

This study aims to test hypotheses about different potential characteristics of members in alternative health groups and the association between membership and attitudes toward vaccination and vaccine-related behaviors.

**Methods:**

A web-based cross-sectional survey (N=1050) was conducted. Participants were recruited from 19 alternative health social media groups and Amazon’s Mechanical Turk. A total of 596 participants were members of alternative health groups and 454 were nonmembers of alternative health groups. Logistic regressions were performed to test the hypotheses about the relationship between membership and the variables of interest.

**Results:**

Logistic regression revealed that there is a positive association between alternative health social media group membership and 3 personal characteristics: sharing trait (B=.83, SE=.11; *P*<.01; odds ratio [OR] 2.30, 95% CI 1.85-2.86), fear of negative evaluations (B=.19, SE=.06; *P*<.001, OR 1.21, 95% CI 1.06-1.37), and conspiratorial mentality (B=.33, SE=.08; *P*<.01; OR 1.40, 95% CI 1.18-1.65). Also, the results indicate that there is a negative association between membership and 2 characteristics: health literacy (B=–1.09, SE=.17; *P*<.001; OR .33, 95% CI 0.23-0.47) and attitudes toward vaccination (B=– 2.33, SE=.09; *P*=.02; OR 0.79, 95% CI 0.65-0.95). However, there is no association between membership and health consciousness (B=.12, SE=.10; *P*=.24; OR 1.13, 95% CI 0.92-1.38). Finally, membership is negatively associated with COVID-19 vaccination status (B=–.84, SE=.17; *P*<.001; OR 48, 95% CI 0.32-0.62), and influenza vaccination practice (B=–1.14, SE=.17; *P*<.001; OR .31, 95% CI 0.22-0.45).

**Conclusions:**

Our findings indicate that people joining alternative health social media groups differ from nonmembers in different aspects, such as sharing, fear of negative evaluations, conspiratorial mentality, and health literacy. They also suggest that there is a significant relationship between membership and vaccination. By more thoroughly exploring the demographic, or by better understanding the people for whom interventions are designed, this study is expected to help researchers to more strategically and effectively develop and implement interventions.

## Introduction

### Background

Social media platforms such as Facebook and Reddit are home to numerous alternative health groups where hundreds and even thousands of people share health issues, learn about strategies to improve their health or achieve their health goals, find support among others, and share alternative health news [1]. Alternative health can be defined as information and treatments that have not been “scientifically researched and consequently approved by professional associations” [2]; examples include homeopathy, naturopathy, and treatments such as chiropractic manipulation [3]. These online communities are also where health and science misinformation are regularly shared among group members or submembers [1]. Health misinformation can be defined as false, misleading, ineffective, and even harmful information and treatments that do “not enjoy universal or near-universal consensus as being true at a particular moment in time on the basis of evidence” [4].

Groups forming around different topics are prominent, and can be promoted by, social media platforms, especially Facebook [5]. These groups, and social media platforms more generally, are important sites for the circulation or diffusion of health information [6] as well as the circulation or diffusion of health misinformation [7]. Alternative health groups, specifically, are a “fertile ground” that engenders “concerns, rumors, and heated debates” [8], which can lead to people refusing critical health care interventions, such as vaccination, and choosing pointless or unsafe medical interventions [9]. This makes social media alternative health groups important spaces for study. Why do people join alternative health groups? What are the characteristics of members of alternative health groups? How does membership in alternative health groups relate to people’s health views or vaccination statuses? While we increasingly understand misinformation as content and how health misinformation circulates across social media platforms [10], we still know very little about who may be likely to join and engage in spaces where health misinformation circulates. So, this study aims to better understand individuals joining alternative health groups on social media and their attitudes toward vaccination and their related behaviors.

### Predisposing Characteristics of Members of Alternative Health Social Media Groups

Individuals are motivated to join online communities for informational and social support [11]. When it comes to health-related virtual communities, the motivations are not different. For example, health patients and caregivers are motivated to join them to obtain or exchange information as well as to get emotional support and empathy from others [12-17]. While sharing patterns might differ depending on the kind of online health communities [18], given the motivations and goals, it is natural for members of online health communities to be information diffusers compared to non-members. Like other online health communities, people might join alternative health groups to share information with other users in the same community. Indeed, research on participants in alternative health groups indicates that members frequently share what they learn about different health topics with other online health group members [1]. Therefore, the following was hypothesized as H1: Membership in alternative health groups on social media is positively associated with online sharing traits.

While people could get and exchange informational and social support offline in the context of health, some individuals choose to join online communities for these types of support, as they see online spaces as “safe places” to connect with others and receive information [19,20]. Online groups might provide those who deal with health-related stigmatization with “safer places” [21] for discussions of topics that might be negatively perceived [22-24]. Given the negative perceptions of alternative health groups on social media [25-27], individuals who are interested in alternative health and would like to obtain or exchange information about alternative health information might need “safer places” as well. Fear of negative evaluations, “apprehension and distress arising from concerns about being judged disparagingly or hostilely by others” [28], is positively associated with the effect of mediated channels, rather than face-to-face interactions [29], these people might go online to interact with others with the same interest. Therefore, the following was hypothesized as H2: Membership in alternative health groups on social media is positively associated with fear of negative evaluations.

Many of the alternative health groups on social media have become spaces for sharing misinformation [1,30]. So, these groups might attract those who are vulnerable to inaccurate health information (ie, those who are more likely to believe inaccurate health information as true). Conspiratorial mentality, the general tendency “to subscribe to theories blaming a conspiracy of ill-intending individuals or groups for important societal phenomena” [31], is one of the contributors to misinformation belief [32]. Also, those with a strong conspiratorial mentality tend to mistrust any official sources [33] so they might rely more on alternative health groups as sources of health information. Therefore, the following was hypothesized as H3: Membership in alternative health groups on social media is positively associated with a conspiratorial mentality.

Health consciousness refers to the degree to which individuals attend to their health [34]. A study on the relationship between health consciousness and health care shows that those who are health-conscious are “more open to unorthodox medical alternatives than less health-conscious people” [35], which implies that highly health-conscious individuals are more likely to be open to a variety of potential health options and nontraditional sources of health information, such as social media groups. Also, health consciousness positively impacts community participation [36], so they may be more motivated to join online health communities, including alternative health groups. Therefore, the following was hypothesized as H4**:** Membership in alternative health groups on social media is positively associated with health consciousness.

Health literacy is another related but different concept that needs to be understood the effect of individual differences on alternative health group membership. Health literacy involves knowledge of health, processing and using health information, and the ability to maintain health by applying the information [37]. Diviani et al [38] found that low health literacy is negatively associated with the ability to evaluate online health information. As misinformation is widely shared in alternative health groups on social media [1,30], those with high levels of health literacy might not join alternative health groups, while those with low levels of health literacy might join them. Therefore, the following is hypothesized as H5: Membership in alternative health groups on social media is negatively associated with health literacy.

### Association Between Membership in Alternative Health Groups and Vaccination

Research has shown that misinformation about vaccines is prevalent on social media [39-41]. As vaccination misinformation focuses on the side effects of vaccination [41-43], it is likely that misinformation on vaccination results in negative attitudes toward vaccination. So, members of alternative health groups are more likely to encounter false claims about vaccination than nonmembers and, in turn, more likely to have negative attitudes toward vaccination. Alternatively, it is possible that those who already have negative attitudes toward vaccination are more likely to join alternative health groups to obtain vaccine information that is consistent with their belief in vaccination and the attitude was reinforced [44]. Therefore, the following was hypothesized as H6: Membership in alternative health groups on social media is negatively associated with attitude toward vaccination.

Vaccine-related misinformation on social media results in vaccination hesitancy [45,46]. Besides, a study reported that COVID-19 vaccination rates are negatively associated with influenza vaccination rates, suggesting that it might be due to lower trust in public health [47]. In other words, attitudes toward COVID-19 vaccination might be associated with attitudes toward influenza vaccination. Therefore, the following was hypothesized as H7 and H8: Membership in alternative health groups on social media is negatively associated with COVID-19 vaccination and influenza vaccination.

This study aims to understand various characteristics of alternative health social media group members by testing the aforementioned hypotheses. Their characteristics would ultimately help develop interventions to curb the spread of health misinformation among members of those groups.

## Methods

### Survey Details

To test the hypotheses, a web-based cross-sectional open survey was conducted, which resulted in a convenience sample of 1050. Data were collected between May 19, 2022, and June 6, 2022. Regardless of the membership status, only 18 years or older living in the United States were eligible to participate. To recruit members of alternative health groups on social media, we identified these spaces for recruitment based on preliminary observations and qualitative interviews conducted for the investigators’ previous studies [1,48]. For the previous studies, we identified these spaces with keywords such as “Alternative Health,” “Natural Health,” and “Holistic Health.” We could not randomly select these groups to invite to participate because all groups are not searchable or public (ie, anyone can search from them and join without requiring permission), all groups are not open to research projects, and some groups are inactive (eg, only one member exists or no post for several months). After we contacted the administrators of active groups, we were able to obtain permission from 15 groups on Facebook and 4 groups on Reddit (refer to Multimedia Appendix 1 for the list). We posted the participant recruitment advertisement to each group as instructed by the group administrator so that participants could voluntarily participate. To recruit nonmembers and members from alternative health groups other than the aforementioned 19 groups, the survey also recruited participants on Amazon’s Mechanical Turk (MTurk).

The survey questions were developed based on previous qualitative studies [1,48]. Through the studies, we identified variables to measure as potential characteristics of alternative health social media group members. The survey involved pilot and full launches. The survey was created and made available through Qualtrics online survey software. Qualtrics’ “prevent multiple submissions” feature determines unique visitors based on cookies.

In total, 1050 responses from members of alternative health groups (n=596) and nonmembers (n=454) were included in the analyses. In total, 427 members were recruited from social media and 169 were from MTurk. 18 nonmembers were recruited from social media and 436 nonmembers were recruited from MTurk. A total of 1198 individuals started the survey. The participation rate was 98.3% (1178/1198) and the completion rate was 89.1% (1050/1178).

### Ethical Considerations

This study was approved by the Merrimack College institutional review board (IRB-FY-21-22-29). The first page of the online survey presented the study information (eg, study purpose, length, procedure, participants’ rights, privacy, and confidentiality protection information) that is designed to help people’s decision to participate in the study. After reading the information, participants indicated to participate by continuing to the next survey page by clicking the next button.

### Measures

#### Membership in Alternative Health Social Media Groups

Participants were asked to select an alternative health group or groups they had joined. More specifically, 20 options, including “other” were presented. Those who selected at least one group listed were coded as “1,” while those who did not select a group were coded as “0.”

#### Sharing

Sharing was measured with a subscale of the maven scale from Boster et al [49]. Five 5-point Likert-type items include, “When I know something about health-related issues, I feel it is important to share that information with others,” “I like to be aware of the most up-to-date health-related information so I can help others by sharing when it is relevant” (1=Strongly disagree, 5=Strongly agree). The items (α=.77) were averaged to create a measure of health consciousness (mean 3.49, SD .78). Every α in the measures section is the Cronbach α.

#### Fear of Negative Evaluations

We measured participants’ fear of negative evaluations with the measure from Carleton et al [28]. Eight 5-point Likert-type items include, “I am afraid that others will not approve of me,” and “I am concerned about other people’s opinions of me” (0=Extremely uncharacteristic of me; 4=Extremely characteristic of me). The items (α=.95) were averaged to create a measure of fear of negative evaluations (mean 2.90, SD 1.14).

#### Conspiratorial Mentality

The conspiratorial mentality was measured with the 5-point Likert-type items from Bruder et al [31]. The items include, “Many very important things happen in the world, which the public is never informed about,” and “Politicians usually do not tell us the true motives for their decisions” (1=Strongly disagree, 5=Strongly agree). The 5 items (α=.84) were averaged to create a measure of conspiratorial mentality (mean 3.19, SD .99).

#### Health Consciousness

Health consciousness was measured with the Health Consciousness Scale by Gould [34]. Three 5-point Likert-type items include, “I reflect about my health a lot,” “I'm very self-conscious about my health,” and “I'm generally attentive to my inner feelings about my health” (1=Strongly disagree, 5=Strongly agree). The items (α=.76) were averaged to create a measure of health consciousness (mean 3.85, SD .80).

#### Health Literacy

The measure of health literacy from Montagni et al [50] was used. Five 4-point scales include, “I compare health information from different sources” and “When I discover new health information I verify if it is true or not” (0=Completely disagree, 3=Completely agree). The items (α=.63) were averaged to create a measure of health literacy (mean 2.17, SD .49).

#### Attitude Toward Vaccination

The vaccination Attitudes Examination scale [51] was used to measure participants’ attitudes toward vaccines. Twelve 6-point items include, “I feel safe after being vaccinated” and “I can rely on vaccines to stop serious infectious diseases” (1=Strongly disagree, 7=Strongly agree). The items (α=.94) were averaged to create a measure of attitude toward vaccination (mean 4.40, SD 0.87).

#### COVID-19 Vaccination Status

Participants were asked to indicate their COVID-19 vaccination status. Those who never had a COVID-19 vaccine were coded as 0 (18.5%, 194/1050), and those who had at least one shot were coded as 0 (81.5%, 856/1050).

#### Influenza Vaccination Behavior

Influenza vaccination behavior was measured with the question, that is “How often do you get an annual flu shot to protect yourself from seasonal influenza?” Those who never got a flu vaccine were coded as 0 (24.0%, 252/1050), and others were coded as 1 (76.0%, 798/1050).

#### Covariates

We controlled for age, sex, and education in our analyses.

## Results

The total sample size is 1050; the mean age of the sample is 37.12 (SD 11.68) and the sample consists of 574 (54.66%) males, 470 (44.77%) females, and 6 (0.57%) intersex individuals. The racial/ethnic distribution was 66.76% (701/1050) White, 9.52% (100/1050) African American, 6.19% (65/1050) Asian American, 16.38% (172/1050) others and 1.14% (12/1050) declined to answer. Refer to Table 1 for the demographic characteristics of the participants.

A logistic regression was performed to test the association between the predisposing characteristics of interest (ie, sharing, fear of negative evaluations, conspiratorial mentality, health consciousness, health literacy, and attitude toward vaccination) and the membership in alternative health groups on social media, controlling for age, sex, and education, to test hypotheses 1 through 6. See Table 2 for descriptive statistics of the measure variables.

The logistic regression model was statistically significant (*χ*^2^_14_=257.5; *P*<.001). The model explained between 21.8% (Cox & Snell *R*^2^) and 29.2% (Nagelkerke *R*^2^) of the variance in membership and correctly classified 71.7% of cases.

As shown in Table 3, among the 6 variables, 5 of them made a unique statistically significant contribution to the model. Specifically, the data supported hypothesis 1 that there is a positive association between the sharing trait and alternative health social media group membership, controlling for the other variables in the model (B=.83, SE 0.11, Wald= 57.78; *P*<.01, OR 2.30, 95% CI 1.85-2.86). Fear of negative evaluation was found to contribute to the model (B=.19, SE 0.06, Wald=8.86; *P*<.001, OR 1.21, 95% CI 1.06-1.37), indicating there is a positive association between fear of negative evaluation and membership. Therefore, Hypothesis 2 was supported. Also, conspiratorial thinking was found to contribute to the model (B=.33, SE 0.08, Wald=15.40; *P*<.001, OR 1.40, 95% CI 1.18-1.65). This suggests that there is a positive association between conspiratorial thinking and membership, supporting Hypothesis 3. As predicted, there is a negative association between health literacy and membership (B=–1.09, SE 0.17, Wald=39.55; *P*<.001, OR .33, 95% CI .23-.47), supporting Hypothesis 5. Finally, it was found that there is a negative association between attitude toward vaccination and membership (B=–2.33, SE 0.09, Wald=5.89; *P*=.02, OR 0.79, 95% CI 0.65-0.95), supporting Hypothesis 6. Unlike the other variables tested (sharing, fear of negative evaluations, conspiratorial mentality, health consciousness, and health literacy), attitudes toward vaccination might not be a predisposing characteristic, as someone’s attitude could be influenced by other alternative group members. In other words, while it is possible that those with negative attitudes toward vaccination are more likely to join alternative health groups on social media, it is also possible that people might have negative attitudes toward vaccination after they join one of those alternative health groups. To test this possibility, a regression was conducted, controlling for age, sex, and education and the results suggested the possibility. There was a statistically significant difference between members and nonmembers (B=–.09, SE 0.17; *P*<.001). Which one is the case, all these results support Hypothesis 6.

However, health consciousness was not found to contribute to the model, indicating there is no significant association between membership and health consciousness (B=.12, SE 0.10, Wald=1.36; *P*=.24, OR 1.13, 95% CI .92-1.38). Therefore, Hypothesis 4 was not supported.

**Table 1:** Demographic characteristics of participants (N=1050).

Characteristic		Participants
Age (years), mean (SD)		37.12 (11.68)
**Sex, n (%)**		
	Male	574 (54.66)
	Female	470 (44.77)
	Intersex	6 (.57)
**Race, n (%)**		
	White	701 (66.76)
	African American	100 (9.52)
	Asian American	65 (6.19)
	Others	172 (16.38)
	Decline to answer	12 (1.14)
**Education, n (%)**		
	No high school	10 (.95)
	High school graduate	114 (10.86)
	Some college	248 (23.62)
	2-year college	156 (14.86)
	4-year college	394 (37.52)
	Postgraduate	128 (12.19)

**Table 2:** Descriptive statistics of measured variables.

Variables		Members (n=596)		Nonmembers (n=454)		All (N=1050)
Sharing, mean (SD)		3.64 (0.63)		3.29 (0.90)		3.49 (0.78)
Fear of negative evaluations, mean (SD)		3.11 (0.97)		2.63 (1.29)		2.90 (.1.14)
Conspiratorial mentality, mean (SD)		3.41 (0.86)		2.90 (1.07)		3.19 (0.99)
Health consciousness, mean (SD)		3.90 (0.74)		3.79 (0.87)		3.85 (0.80)
Health literacy, mean (SD)		2.10 (0.49)		2.26 (0.49)		2.17 (0.49)
Attitude toward vaccination, mean (SD)		4.26 (0.03)		4.58 (0.93)		4.4 (0.87)
**COVID-19 vaccination status, n (%)**						
	Never vaccinated	75 (12.6)	119 (26.2)		194 (18.5)	
	Others	521 (87.4)	335 (73.8)		856 (81.5)	
**Influenza vaccination behavior, n (%)**						
	Never vaccinated	85 (14.3)	167 (36.8)		252 (24)	
	Others	511 (85.7)	287 (63.2)		798 (76)	

**Table 3:** Logistic regression predicting the likelihood of being a member of alternative health social media groups.

	B	SE	Wald	df	Significance (*P*)	OR (95% CI)
Sharing	.836	0.110	57.789	1	.003	2.306 (1.859-2.861)
Fear of negative evaluations	.190	0.064	8.868	1	.000	1.210 (1.067-1.371)
Conspiratorial mentality	.337	0.086	15.409	1	.000	1.401 (1.184-1.658)
Health consciousness	.123	0.105	1.365	1	.24	1.131 (0.920-1.389)
Health literacy	–1.097	0.174	39.553	1	.000	0.334 (0.237-0.470)
Attitude toward vaccination	–.233	0.096	5.894	1	.02	0.792 (0.656-0.956)

Another logistic regression was performed to examine the relationship between membership and COVID-19 vaccination status (H7). Age, sex, and education were entered into the model as control variables.

The logistic regression model was statistically significant (*χ*^2^_4_=45.8; *P*<.001). The model explained between 4.3% (Cox & Snell *R*^2^) and 6.9% (Nagelkerke *R*^2^) of the variance in membership and correctly classified 81.5% of cases. In the model, membership in alternative health groups on social media is a significant predictor of COVID-19 vaccine status (B=–.84, SE 0.17, Wald=22.36; *P*<.001). The OR is .48 (95% CI .32-.62), indicating that members are .48 times less likely to get at least one dose of the COVID-19 vaccine. The model demonstrates that this is a negative association between membership and COVID-19 vaccination status. Therefore, Hypothesis 7 was supported and Snell *R*^2^).

To Hypothesis 8, a separate logistic regression was run with the same control variables. The model was statistically significant (χ^2^_5_= 45.8; *P*<.001). The model explained between 2.3% (Cox & Snell *R*^2^) and 3.4% (Nagelkerke *R*^2^) of the variance in membership and correctly classified 81.8% of cases. In the model, there is a negative association between membership in alternative health groups on social media and influenza vaccination practice (B=–1.14, SE 0.17, Wald=41.56; *P*<.001). The OR is .31 (95% CI 0.22-0.45), indicating that members are .31 times less likely to get the flu vaccine. The model demonstrates that this is a negative association between membership and general flu vaccination practice, supporting hypothesis 8.

## Discussion

### Summary of Results

This study aimed to better understand members of alternative health groups on social media by looking at predisposing characteristics of members versus nonmembers. The survey results suggest that the online sharing trait is positively associated with membership in alternative health social media groups (H1), fear of negative evaluations is also positively associated with membership (H2), and conspiratorial thinking is positively associated with membership (H3). While there is no significant relationship between health consciousness and membership (H4), there was a negative association between health literacy and membership (H5).

As vaccine-related misinformation is prevalent [39-41], the current survey also examined whether membership in alternative health groups on social media is associated with attitudes toward vaccination and COVID-19 and influenza vaccine-related behaviors. We found that membership is negatively associated with attitudes toward vaccination; members have more negative attitudes than nonmembers. Also, there was a negative association between membership in alternative health groups and COVID-19 vaccine-related behaviors and influenza vaccine practice.

### Implications

These findings help us understand the predisposing characteristics and vaccine-related attitudes and behaviors of people joining alternative health groups where health misinformation circulates. By analyzing the people who participate in these alternative health social media groups, we can better understand members of those groups, engaging with and sharing health misinformation, which is critical to developing both health communication interventions and health interventions. A meta-analysis of studies on health misinformation interventions finds that while interventions can be effective, “the average effect of correction is of weak-moderate magnitude” [52]. A systematic literature review of health misinformation interventions comes to the same conclusion: we still have a limited understanding of how to mitigate health misinformation effectively [53].

We contend that by more thoroughly exploring the who, or by better understanding the people for whom interventions are designed, it will be easier for researchers to more strategically and effectively use interventions. In addition, by analyzing who engages with and shares health misinformation, especially in spaces where health misinformation thrives, we may also be able to address or mitigate some of the underlying conditions that lead people to health misinformation, or that enable the spread and influence of health misinformation, including antivaccine misinformation. This might reduce the need to correct discrete yet continually emerging health misperceptions across social media platforms.

### Limitations and Future Directions

Although this study is a cross-sectional survey, it is legitimate to test the logistic regression models with the variables of interest as possible predictors of membership in alternative health groups on social media because most of them are predisposing characteristics. Yet, our models do not imply causality and should be considered alongside other potential factors affecting individuals’ choice to join alternative health groups on social media.

Also, given that most of the nonmembers were recruited from MTurk, possible sampling bias would be another limitation. Like other studies, there is a need to replicate this study with different samples. Research has shown some demographic differences, such as age, sex, and education, between MTurk “workers” and nationally representative samples [54-57]. So, we controlled for them in our analyses as recommended [58], although other existing research suggests they are close to the general population regarding demographic characteristics [59]. Yet, to avoid any possible sampling bias and test the hypotheses in a more robust way, for replication studies, using representative samples would be ideal.

Additional research is needed to further identify and explore the thoughts and beliefs, tendencies, behavioral intentions, and other characteristics of people who are likely to engage in spaces rife with health misinformation. More research is also needed to interrogate how participants in these alternative health groups find information, assess whether they trust or distrust information, and determine factors that go into whether and why they share information within and beyond the social media groups to which they belong. As previously mentioned, there is a great need to determine the most effective and scalable ways to not only address health and science misinformation, but also to contend with the underlying factors and contexts that can make people more likely to receive, engage with, or share health misinformation, including antivaccine misinformation. To do any of this, we need to focus more on who shares misinformation, and why they share misinformation, as much as we do on what kinds of health misinformation is shared and how it spreads.

## Appendix

Multimedia Appendix 1 Survey questionnaire.

## Abbreviations

MTurk: Amazon’s Mechanical Turk

## Data Availability

The data sets generated during and/or analyzed during this study are available from the corresponding author on reasonable request.
