# Supplementary material for: Understanding Membership in Alternative Health Social Media Groups and Its Association with COVID-19 and Influenza Vaccination: Web-Based Cross-Sectional Survey
Source: JMIR Form Res. 2024 Dec 5;8:e54092. doi: 10.2196/54092 (PMC11659688; doi:10.2196/54092)
Supplement: Multimedia Appendix 1 [file formative_v8i1e54092_app1.docx]

# **Survey Questionnaire**

## **Screening Questions**

*Note: Individuals 18 or older living in the U.S. were eligible to participate in this study.*

1. How old are you?
2. Are you currently living in the U.S.?
   - - Yes
     - No

**[Consent Form Presented Here]**

## **Survey Questions**

*Note: The names of variables measured are in brackets and the names were not shown to participants.*

1. [Alternative health social media group membership] There are many social media groups for health/science information. Are you joining any of those groups? Please select all that apply.
   - 1. Facebook – *Covid Early Treatment Experiences (Ivermectin, etc.)*
     2. Facebook – *Friends who like Dr. Tenpenny on Vaccines*
     3. Facebook – *Health News*
     4. Facebook – *Health Nut*
     5. Facebook – *Dr. Tenpenny*
     6. Facebook – *Medical Medium Northeast USA*
     7. Facebook – *Alternative and Natural Health*
     8. Facebook – *Health and news*
     9. Facebook – *Health News 24x7 (Health Tips, Food Recipes, Fitness, Diet & Nutrition)*
     10. Facebook – *Medical Medium Intuitive, Herbal-Homeopathic & Food as Medicine*
     11. Facebook – *Health Tips and News*
     12. Facebook – *Health Tips and Recipes*
     13. Facebook – *Health and Fitness*
     14. Facebook – *The Holistic Approach (Root Cause Concept)*
     15. Facebook – *Covid-19 vaccine side effects group (Pfizer, Moderna, Astra, Johnson,..)*
     16. Reddit – *Covid-19*
     17. Reddit – *LockdownSkepticism*
     18. Reddit – *Alternative Healthy Living*
     19. Reddit – *Debate Vaccines*
     20. Other [Specify the platform and the name of the group]
     21. None

*Note: The study participant recruitment ads were posted to Groups 1-19 listed above.*

1. [Fear of negative evaluation [1]] Please tell us how well each of the following statements characterizes you (0 = *Extremely uncharacteristic of me*; 4 = *Extremely characteristic of me*).
   - - I am frequently afraid of other people noticing my shortcomings.
     - I am afraid that others will not approve of me.
     - I am concerned about other people's opinions of me.
     - When I am talking to someone, I worry about what they may be thinking about me.
     - I am usually worried about what kind of impression I make.
     - If I know someone is judging me, it tends to bother me.
     - I often worry that I will say or do wrong things.
     - I worry about what other people will think of me even when I know it doesn't make any difference.
2. [Conspiratorial mentality [2]] I think…(1 = *Strongly disagree*, 5 = *Strongly agree*)
   - - Many very important things happen in the world, which the public is never informed about.
     - Politicians usually do not tell us the true motives for their decisions.
     - Government agencies closely monitor all citizens.
     - Events which superficially seem to lack a connection are often the result of secret activities
     - There are secret organizations that greatly influence political decisions.
3. [Health literacy; Used in Montagni, Ouazzani-Touhami, Mebarki, Texier, Schück, Tzourio, and group[3]] Please indicate the extent to which you agree with each of the following statements (0 = *Completely disagree*, 3 = *Completely agree*)
   - - I compare health information from different sources
     - When I discover new health information I verify if it is true or not
     - I decide what health information is best for me
     - I can state if health information is adapted to my situation or not
     - I enquiry health professionals on the quality of information I find
4. [Health consciousness; Subscale of Gould[4]] Please indicate the extent to which you agree with each of the following statements. (1= *Strongly disagree*, 5 = *Strongly agree*)
   - - I reflect about my health a lot.
     - I'm very self-conscious about my health.
     - I'm generally attentive to my inner feelings about my health
5. [Sharing; Revised Subscale of maven [5]] Please indicate the extent to which you agree with each of the following statements. (1= *Strongly disagree*, 5 = *Strongly agree*)
   - - When I know something about health-related issues, I feel it is important to share that information with others
     - I like to be aware of the most up-to-date health-related information so I can help others by sharing when it is relevant
     - If someone asked me about a health-related issue that I was unsure of, I would know how to help them find the answer
     - Being knowledgeable enough about health-related issues so that I could teach someone else is important to me
     - People often seek me out for answers when they have questions about a health-related issue
6. [Attitude toward vaccination; Vaccination Attitudes Examination (VAX) scale [6]; (R): Reversed item] Please indicate the extent to which you agree with the following statements (1 = *Strongly disagree*, 6 = *Strongly agree*)
   - [Subscale: Mistrust of vaccine benefit]
     - I feel safe after being vaccinated (R)
     - I can rely on vaccines to stop serious infectious diseases (R)
     - I can rely on vaccines to stop serious infectious diseases (R)
   - [Subscale: Worries about unforeseen future effects]

- Although most vaccines appear to be safe, there may be problems that we have not yet discovered.
- Vaccines can cause unforeseen problems in children.
- I worry about the unknown effects of vaccines in the future.
  - [Subscale: Concerns about commercial profiteering]
    - Vaccines make a lot of money for pharmaceutical companies, but do not do much for regular people.
    - Authorities promote vaccination for financial gain, not for people's health.
    - Vaccination programs are a big con.
  - [Subscale: Preference for natural immunity]
    - Natural immunity lasts longer than a vaccination.
    - Natural exposure to viruses and germs gives the safest protection.
    - Being exposed to diseases naturally is safer for the immune system than being exposed through vaccination.

1. [COVID-19 vaccination status] Have you been vaccinated to protect yourself from COVID-19?
   - - Yes, I have had one shot
     - Yes, I am fully vaccinated (two shots)
     - Yes, I have had a booster shot
     - No, I have not received a shot
2. [Flu vaccination] How often do you get an annual flu shot to protect yourself from seasonal influenza?
   - - Never
     - Rarely
     - Some years
     - Most years
     - Every year

## **Demographic Questions**

1. Sex
   - - Male
     - Female
     - Intersex
2. Race [Select all that apply]
   - - American Indian or Alaska native
     - Asian
     - Black or African American
     - Native Hawaiian or other Pacific Islander
     - White
     - Other [Specify]
3. Ethnicity
   - - Hispanic Origin
     - Not of Hispanic Origin
4. Education
   - - No high school
     - High school graduate
     - Some college
     - 2-year college
     - 4-year college
     - Post-graduate

## **References**

1. Carleton RN, Collimore KC and Asmundson GJG. Social anxiety and fear of negative evaluation: Construct validity of the BFNE-II. Journal of Anxiety Disorders. 2007/01/01/ 2007;21(1):131-141. doi:10.1016/j.janxdis.2006.03.010
2. Bruder M, Haffke P, Neave N, Nouripanah N and Imhoff R. Measuring individual differences in generic beliefs in conspiracy Theories across cultures: Conspiracy mentality questionnaire. Frontiers in Psychology. 2013;4(225)doi:10.3389/fpsyg.2013.00225
3. Montagni I, Ouazzani-Touhami K, Mebarki A, et al. Acceptance of a Covid-19 vaccine is associated with ability to detect fake news and health literacy. Journal of Public Health. 2021;43(4):695-702. doi:10.1093/pubmed/fdab028
4. Gould SJ. Health consciousness and health behavior: The application of a new health consciousness scale. American Journal of Preventive Medicine. 1990;6(4):228-237. doi:10.1016/S0749-3797(18)31009-2
5. Boster FJ, Carpenter CJ and Kotowksi MR. Validation studies of the maven scale. Social Influence. 2015;10(2):85-96. doi:10.1080/15534510.2014.939224
6. Martin LR and Petrie KJ. Understanding the dimensions of anti-vaccination attitudes: the Vaccination Attitudes Examination (VAX) scale. Annals of Behavioral Medicine. 2017;51(5):652-660. doi:10.1007/s12160-017-9888-y
